# Response to comment on: Is “pre-sepsis” the new sepsis? A narrative review

**DOI:** 10.1371/journal.ppat.1013952

**Published:** 2026-03-06

**Authors:** Rémy Gerard, Antoine Dewitte, Fridolin Gross, Thomas Pradeu, Maël Lemoine, Julien Goret, Maria Mamani-Matsuda

**Affiliations:** 1 Department of Pediatrics, Neonatal Intensive Care Unit, Bordeaux University Hospital, Bordeaux, France; 2 University of Bordeaux, ImmunoConcEpT, CNRS UMR 5164, Inserm ERL 1303, Bordeaux, France; 3 Department of Anaesthesia and Intensive Care, Bordeaux University Hospital, Pessac, France; 4 University of Bordeaux, Immuno ConcEpT, CNRS UMR 5164, Bordeaux, France; 5 Department of Immunology and Immunogenetics, Bordeaux University Hospital, Bordeaux, France; INSERM, FRANCE

## Dear Editor

We appreciate the commentary from Giglio and associates, and found it highly engaging. Their real-world experiences provides valuable practical insight alongside the theoretical framework we previously outlined earlier regarding pre-sepsis.

The described methodology, based on artificial intelligence and machine learning, enables the identification of distinct patient phenotypes before the onset of sepsis. The “progressive pre-sepsis” and “aborted sepsis evolution” phenotypes align well with a dynamic conceptual model of pre-sepsis [1]. The former illustrates a trajectory in which host compensatory mechanisms initially preserve homeostasis but are ultimately overwhelmed, culminating in sepsis. This finding underscores the potential to intervene within a critical time frame to support functional reserves and maintain physiological stability. The latter phenotype further supports that timely recognition and intervention during this window may interrupt the trajectory and prevent progression to overt sepsis.

At the same time, we agree that infections that do not progress to sepsis should not be classified as pre-sepsis. Many infected patients—especially those with preserved physiological reserves—mount an effective, self-limited response and never reach the dysregulation phase that characterizes sepsis. Giglio et al. also discuss an intermediate clinical group they call “sub-threshold sepsis patients” in which physiological dysregulation and early organ dysfunction may be emerging despite not meeting official criteria for sepsis [2]. As noted by the authors, this highlights a limitation of cutoff-based definitions: patients may be deteriorating through modest changes across multiple continuous variables while remaining below formal thresholds. In such cases, this state may be closer to early sepsis than to pre-sepsis, underscoring the need to better align terminology with underlying dynamics.

This is precisely where a discontinuity framework may add value. Sepsis can be conceptualized as a nonlinear shift in system behavior occurring when functional reserves are exhausted and compensatory networks fail, leading to rapid decompensation. In complex systems, early-warning signals such as reduced physiological variability and slower recovery after perturbations may precede the tipping point [3]. AI approaches are well suited to integrate high-dimensional clinical time-series and detect proximity to this transition; however, interpretability, transportability, and multicenter validation remain essential to ensure clinical trust and generalizability.

In parallel, we advocate a complementary, trigger-centered strategy aimed at prospectively capturing the earliest host–pathogen encounter and relating infectious triggers to the evolving host-response landscape. Beyond supporting clinical decisions, this perspective could help distinguish tolerated microbial signals from those that precipitate destabilization, and better link “what happens first” to “who decompensates and why”—in other words, linking triggers, functional reserve, and trajectory.

Overall, the work by Giglio et al. is an important contribution that strengthens the clinical plausibility of pre-sepsis. The next step will likely require converging approaches: AI-supported trajectory recognition, mechanistic biomarkers of early dysregulation (including those related to functional reserve), and prospective multicenter studies testing whether intervening during this window can prevent the discontinuous transition to overt sepsis.
